# Balancing hope and uncertainty: family perspectives on lung transplantation in cystic fibrosis – a qualitative study

**DOI:** 10.1080/17482631.2026.2620417

**Published:** 2026-01-24

**Authors:** Ulrika Skogeland, Isabelle de Monestrol, Tove Godskesen

**Affiliations:** a CLINTEC, Department of Paediatrics, Karolinska Institutet, Stockholm, Sweden; b Cystic Fibrosis Centre, Karolinska University Hospital, Stockholm, Sweden; c Nord University, Faculty of Nursing and Health Sciences, Bodø, Norway; d Centre for Research Ethics & Bioethics, Department of Public Health and Caring Sciences, Uppsala University, Uppsala, Sweden

**Keywords:** Cystic fibrosis, family members, lung transplantation, semi-structured interviews, qualitative research

## Abstract

**Purpose:**

Cystic fibrosis (CF) is a genetic disease primarily affecting the lungs and digestive system. Individuals with advanced CF lung disease may require transplantation to survive. Family members often take on significant caregiving roles, facing both emotional and practical challenges throughout the transplantation process. This study explores the experiences of such family members to inform and improve supportive care practices.

**Method:**

Employing a naturalistic, exploratory design, this qualitative study used purposive sampling to recruit 19 family members of lung transplant recipients with CF. Data were collected through semi-structured interviews and analysed using reflexive thematic analysis.

**Results:**

The analysis identified three main themes and eight subthemes: (I) balancing hope and despair on the waiting list, (II) navigating challenges and finding relief after the transplantation, and (III) unmet support and informational needs before and after transplantation.

**Conclusion:**

This study highlights the emotional burden and caregiving responsibilities shouldered by family members of individuals with CF who have undergone lung transplantation. The findings emphasise the importance of person- and family-centred interventions, including support for palliative care discussions. A more structured and inclusive framework is essential to address the often-overlooked needs of families throughout the transplantation process.

## Background

Cystic fibrosis (CF) is a severe genetic disorder that significantly impacts the respiratory and digestive systems, Cystic fibrosis (CF) is primarily caused by mutations in the cystic fibrosis transmembrane conductance regulator (*CFTR*) gene (Rafeeq & Murad, 2017; Shteinberg et al., 2021). This condition leads to the accumulation of thick, sticky mucus in various organs, particularly the lungs. Over time, this leads to progressive lung damage and a notable reduction in life expectancy for affected individuals.

Recent advancements in medical treatment, particularly the development of CFTR modulators, have significantly improved life expectancy and quality of life for many individuals with CF. CFTR modulators are pharmacological therapies that target the underlying defect in CFTR protein, improving its function and thereby addressing the root cause of CF. However, these therapies are not universally effective. They cannot be used by individuals with rare mutations or by those who experience severe side effects, and advanced lung disease may still progress; these individuals may ultimately require lung transplantation (Burdis et al., 2024; Middleton & Taylor-Cousar, 2021; Yeung et al., 2020). In cases where respiratory insufficiency becomes critical, lung transplantation may be the only viable option to prolong life (Rafeeq & Murad, 2017; Shteinberg et al., 2021).

The impact of CF extends beyond those diagnosed, placing a significant burden on family members, who frequently assume informal caregiving roles. These caregivers are integral to daily disease management, providing both emotional support and practical assistance (Wojtaszczyk et al., 2018; Yagelniski et al., 2020). However, the responsibilities associated with caregiving can lead to considerable physical, emotional, and psychological strain, adversely affecting the caregivers’ overall well-being and quality of life (Fitzgerald et al., 2018; Quittner et al., 2014).

Lung transplantation introduces a distinct set of emotional and practical demands, further increasing the responsibilities of family members involved in maintaining the well-being and care of individuals with CF (Yagelniski et al., 2020). While the medical and physiological aspects of CF have been extensively studied (Fitzgerald et al., 2018), the experiences and needs of family caregivers during lung transplantation remain underexplored (Yagelniski et al., 2020).

Understanding family members’ coping strategies, emotional burdens, and unmet needs can guide support interventions that benefit family members and potentially improve outcomes for individuals with CF (Deng et al., 2023). This study aims to explore the experiences of family members supporting individuals with CF through the lung transplantation process, with the goal of informing care practices to improve supportive care.

## Method

### Design

This study utilised a naturalistic and exploratory qualitative design (Polit & Beck, 2021). In line with Braun and Clarke’s perspective and Denzin and Lincoln’s definition of qualitative research as creative, reflexive, and subjective, we embraced the researcher’s subjectivity as an essential resource in the research process (Braun & Clarke, 2022; Denzin & Lincoln, 2018). The study follows the Consolidated Criteria for Reporting Qualitative Research (COREQ) checklist for reporting qualitative research (Tong et al., 2007).

### Setting

The study was conducted across two of the four specialised CF Centres located within university hospitals in Sweden. CF centres collectively provide comprehensive long-term care and enhancement of quality of life to approximately 800 individuals living with CF through the implementation of individualised treatment plans.

Lung transplantations are performed exclusively at two transplantation centres in Sweden. During the first year after lung transplantation, care is primarily managed by the transplantation centre, with a focus on recovery, adjustment of immunosuppressive therapy, and monitoring for complications. Thereafter, responsibility for long-term follow-up shifts to the CF centres.

The two CF Centres included in this study did not have a transplantation centre in the same city; consequently, all participating family members supported individuals who had to travel long distances to another city for transplantation-related care. This geographical separation may complicate care coordination and place additional logistical and emotional demands on families. This situation underscores the importance of effective collaboration and communication between the CF and transplantation centres to ensure continuous and person-centred care.

### Recruitment and participants

A purposive sampling strategy was employed for participant recruitment. In a previous study conducted by Skogeland et al. (2025), individuals with CF shared their experiences related to lung transplantation. As part of the present research initiative, participants from that study were asked for their consent to the contacting of family members aged 18 years or older for inclusion in the current study, which focuses on the experiences of family members. This request was made approximately one year after the original interviews with individuals with CF (Skogeland et al., 2025), and the procedure was conducted after ethics committee approval. In total, 20 individuals with CF consented and provided contact information for the family members they preferred to have contacted.

All presumptive participants were contacted and informed about the study’s aim via telephone or mail, subsequently receiving written information by mail. Participants were given adequate time to consider their involvement and were encouraged to pose any questions. They were explicitly informed that participation was voluntary and that they had the right to withdraw from the study at any time without the need to provide justification.

### Data collection

An interview guide was developed for this study. The initial version, collaboratively drafted by US and TG, was reviewed by healthcare professionals with clinical experience in CF care, including a registered nurse (RN), a social worker, and a psychologist. Additionally, the guide was evaluated by two individuals with CF who had previously undergone lung transplantation, as well as a family member of an individual with CF who also participated in this study. The feedback received from these stakeholders prompted several refinements to the questions included in the guide. The final, post-review version of the interview guide is presented in Table I.

**Table I.** Interview guide.

Question
How long did your partner/child/parent wait for a lung transplantation? How did your family experience the waiting period?
Can you walk us through your family’s experience with the decision-making process for the lung transplantation? What were the key factors or discussions?
What were your family’s most significant challenges during the waiting period? How did you navigate those challenges together?
What kind of support was most helpful to you and your family during this time? Were there specific types of support you felt were missing?
Did your family discuss sensitive topics, such as risks or difficult decisions? If so, how did you approach these discussions?
Looking back, is there anything you wish had been handled differently during the transplantation process?
Was the information provided by the healthcare team clear, concise, and timely? Did the information help you feel informed and prepared?
Did you feel that healthcare professionals respected your family’s cultural, religious, and personal beliefs? If not, what could have been done differently?
Can you share how life has changed for your family since the transplantation? What has been the most significant adjustment for everyone?
What advice would you give to CF Centre healthcare professionals to better support families during the transplantation process? Are there specific recommendations you would like to share based on your experience?

Prior to data collection, the author US carried out a pilot study with two healthcare professionals in CF care. The aim was to assess the comprehensiveness and applicability of the interview guide. The interviews from the pilot study were not included in the study.

Data collection occurred from January through February 2024. The interviews were conducted in various settings according to participants’ preferences, including in a quiet room at the hospital (*n* = 3), via phone call (*n* = 3), and through the digital Teams platform (*n* = 13). The duration of the interviews ranged from 26 to 87 minutes. Each session was audio recorded and subsequently transcribed verbatim by professional transcriptionists. To ensure confidentiality and promote an atmosphere of transparent and open communication, only the participants and US were present during the interviews.

### Data analysis

The narratives of participants were analysed using reflexive thematic analysis as outlined by Braun and Clarke (2022). This approach encompasses six iterative steps: 1) becoming familiar with the data, 2) generating codes, 3) developing themes, 4) reviewing themes, 5) refining and defining themes, and 6) producing the final report.

During the familiarisation phase, US engaged with each interview multiple times, taking detailed notes on initial impressions and highlighting relevant sections within the texts. Subsequently, US and TG collaboratively generated codes by identifying patterns and organising the data in alignment with the study’s objectives.

Following the coding process, similar codes were organised and grouped to identify potential themes and subthemes, with emphasis on experiences shared by multiple participants. The data management involved collecting codes and sorting them into Excel columns based on similarities to facilitate the detection of broader patterns.

In the subsequent stages, US and TG refined the themes by reassessing their alignment with the coded data. All themes were reviewed for coherence, ensuring conceptual distinctiveness and fidelity to participants’ narratives. Finally, all authors independently reviewed and discussed the themes to achieve consensus before finalising the thematic structure and draughting the findings.

### Ethical approval and consent to participate

In compliance with Swedish legislation concerning research involving human subjects (Act (SFS 2022:49) concerning the Ethical Review of Research Involving Humans), formal ethical approval was obtained from the Swedish Ethical Review Authority, an independent national authority not affiliated with any of the authors. This study received approval from the Swedish Ethical Review Authority (reference number 2023-06374-01). Furthermore, institutional approval was granted by the medical unit directors and section heads at the participating CF Centres (diary number K 2023-9861).

The research adhered to the ethical principles outlined in the Declaration of Helsinki (World Medical Association [WMA], 2024) and complied with the EU Data Protection Regulation (EU, 2016, p. 679).

Before the interviews began, all participants provided written informed consent. They were informed about the study’s purpose, their right to withdraw at any time without any repercussions, and the strict confidentiality of their data. They were also assured that all personal information would be protected.

All personal identifiers were excluded during transcription, and each interview was replaced with a unique pseudonymized code to protect privacy and confidentiality. The code key was securely stored on password-protected devices and kept entirely separate from the interview material. For participants, these codes begin with “P” and are followed by a number ranging from 1 to 19.

## Results

The study included interviews with 19 participants, all of whom were family members of individuals with CF who had undergone lung transplantation and were alive at the time of the interviews (Table II).

**Table II.** Characteristics of family members (n = 19).

Variables	*n* (%) or range
Gender	
Women (*n*)	14 (73.6)
Men (*n*)	5 (26.4)
Relationship with the individual with CF	
Wife/husband/partner	9 (47.3)
Daughter	4 (21.0)
Mother	5 (26.3)
Sister	1 (5.3)
Age in years (median)	26–75 years (52)
Duration variables	
Time on the waiting list	8 days–30 months
Time since lung transplantation	2–20 years

The analysis generated three overarching themes, each encompassing multiple subthemes that reflect emotional, relational, and informational challenges throughout the transplantation process (Table III).

**Table III.** Themes and Subthemes.

Theme	Subthemes
Balancing hope and despair on the waiting list	*Finding control amid the uncertainty* *Important conversations about sensitive topics* *Relationships under strain* *The call–A moment of hope and fear*
Navigating challenges and finding relief after the transplantation	*Balancing social interactions and restrictions* *Notable changes in everyday life*
Unmet support and informational needs before and after transplantation	*A critical need for information and comprehensive emotional support* *A desire to connect with other families involved in the transplantation process*

### Balancing hope and despair on the waiting list

Families described the waiting period as emotionally intense and dominated by the tension between hope and despair. The possibility of lung transplantation brought initial optimism, but as waiting times extended, this hope eroded under the weight of uncertainty, fear of loss, and emotional exhaustion.

The strain influenced daily routines and family dynamics. End-of-life conversations were both necessary and emotionally fraught. Some families addressed them directly, while others avoided them altogether, often with long-term emotional consequences. Emotional resilience was tested both by the wait itself and by the guilt associated with knowing that the life-saving transplantation depended on another person’s death.

#### Finding control amid the uncertainty

The long and uncertain wait for a lung transplantation generated constant emotional tension and overwhelming stress, as family members lived in a state of suspended time, never knowing when their lives might change in an instant.

Many described the emotional toll of struggling with unanswered questions about the health of the individual with CF. The relentless waiting created a sense of emotional exhaustion and a feeling of being trapped in a situation beyond their control. One participant observed:

*“The wait was horrible; I was alone, lost, sad, chaotic, and struggling with my sister. I did not feel well at all. We felt isolated as Dad became sicker.”* (P3)

In response, many participants sought to impose structure on the chaos. Practical strategies such as keeping bags packed, organising medical paperwork, or adhering to fixed routines created a fragile sense of readiness. Emotional support also played a central role. One participant noted:

*“I found support in talking to my colleagues about what we were going through. They listened and offered their support, which made me feel less alone in this difficult time.”* (P6)

Activities like cooking together or watching movies helped families reclaim brief moments of normalcy, offering small reprieves from the emotional strain.

#### Important conversations about sensitive topics

Facing the possibility of death prompted participants to consider conversations they had never wanted to have. For some, openly addressing the risk of loss was a way of preparing emotionally, even when such conversations were heartbreaking. A participant described the actions of her husband, who was on the waiting list:

*“He wrote a letter for our daughter, intended to be read when she grew older. As of now, that letter remains unopened.”* (P19)

This captures the painful intersection of love, preparation, and the profound uncertainty of their situation. Another participant described their experience of addressing sensitive topics directly.

*“We talked about everything … planned her funeral, funding for her child, and talked about pain management if she would become incapable of doing that.”* (P10)

Others, however, chose avoidance as a coping strategy, hoping that focusing on the future would shield them from pain. This avoidance, though protective in the moment, often led to regret:

*“Looking back, I realise that was a mistake, and I have been working through those feelings ever since.”* (P2)

#### Relationships under strain

The emotional weight of caregiving and the prolonged uncertainty during the transplantation process placed considerable strain on family relationships. Partners, in particular, often felt overwhelmed by the demands of being both emotional and practical anchors for their families. One participant described how caregiving responsibilities, work, and emotional pressure converged into a relentless routine:

*“I found myself living an entirely different life … as I was burdened with many responsibilities.”* (P8)

Another reflected on the impossibility of rest, explaining:

*“[I] could not be away for extended times as I had no additional help. I had to work full time because she [the partner] could not work. I even felt the pressure to be perfect at my job.”* (P6)

Children also struggled emotionally, especially during extended hospitalisations, when parents’ absence led to fear, loneliness, and confusion. One participant recalled being told, as a child, that their mother would be gone only briefly, only to feel abandoned when she did not return for weeks:

*“I felt so sad, lonely and frightened.”* (P17)

These prolonged separations disrupted their emotional stability and created uncertainty in their everyday lives.

Siblings, too, experienced emotional conflict, particularly when attention was focused almost entirely on the individual with CF:

*“I often found myself overshadowed [in my childhood] when my sister was put on a pedestal. I have loved her above all else, and there is nothing wrong with that, but it made me feel jealous at times because she received so much attention from everyone.”* (P10)

These emotions, while difficult, were often silently endured by participants, who rarely voiced their feelings amid the family’s focus on the individual with CF. As participants reflected on these strained relationships, many recognised that the impact of CF extended far beyond the medical. The condition shaped the family dynamic in enduring ways, some of which became fully apparent only later. One participant shared:

*“Now that my father has been transplanted, I see how completely the disease defined our family life.”* (P2)

This underscores how CF caregiving, while centred on one individual, affects the entire family unit, leaving emotional imprints that persist long after the transplantation itself.

#### The call—A moment of hope and fear

The anticipation of the transplantation call became a central part of daily life for many participants. Days were marked by a constant sense of readiness, with bags packed by the door and phones never out of reach. This state of heightened alertness was emotionally draining, and as time went on, some began to fear that the call would never come. One participant recalled:

*“The bags stood there, waiting for the much-anticipated phone call, but I began to suspect that we would not receive the call in time … but then, one Tuesday, the call finally came.”* (P6)

When the call finally did arrive, it brought an overwhelming wave of emotions—relief, disbelief, and fear, all at once. The moment, though long-awaited, felt surreal after so many months of waiting and emotional strain. It marked a clear shift from passive endurance to active change, thrusting participants into a new phase without time to fully process what was unfolding:

*“And so, one night … or late evening … the call came, and we had our bags and everything ready. My wife answered and said, “I am ready.” It was no problem, but then time stood still, and now it was for real. And you were both relieved and scared at the same time.”* (P1)

### Navigating challenges and finding relief after the transplantation

Transplantation brought visible improvements in health and a sense of renewed hope for the future. As the immediate medical demands lessened, daily life gradually became less defined by illness. Yet, this relief was accompanied by new emotional and practical challenges, including isolation, ongoing fear of infection, and unresolved feelings such as guilt. These experiences highlighted the fact that while transplantation marked a turning point, it did not mark the end of the process.

#### Balancing social interactions and restrictions

Although lung transplantation offered a renewed sense of health and stability for individuals with CF, it also introduced new limitations and concerns for those around them.

Participants described the period after transplantation as one of constant negotiation between protecting the transplanted lung from infection and maintaining some sense of normalcy in their lives. The need for ongoing precautions, such as avoiding crowds and limiting children’s social contact, reshaped family routines and strained social relationships. This balancing act was not only practical but also deeply emotional, as participants navigated competing responsibilities and lingering fear. One mother of a child whose father had CF described the impact on her family:

*“Our children cannot go to daycare during infection outbreaks, forcing them to stay home and affecting their well-being. They don’t understand why they can’t go like the other kids, and I constantly feel torn between protecting my husband and trying to give them a normal childhood.”* (P19)

The protective choices, while necessary, left participants feeling conflicted, isolated, and stretched thin. For many, the fear of causing harm was ever-present. As one daughter shared:

*“I am terrified of unintentionally giving my father any infections, which makes me afraid to be around him.”* (P3)

Social lives also shifted dramatically. Friends drifted away, invitations became scarce, and spontaneity disappeared from daily life, as reported by another daughter, who remarked:

*“I had to adjust to new routines and felt more isolated. Friends stopped inviting me after a while because I always had to say no. I couldn’t go to crowded places or do spontaneous things anymore, and it felt like my life was limited.”* (P17)

#### Notable changes in everyday life

Following transplantation, participants described a profound shift in their daily lives from routines dominated by illness and constant care to a renewed sense of freedom, stability, and possibility. Although concerns about infection never entirely disappeared, the physical transformation of the transplanted person brought visible relief and hope.

Participants noted how medical routines that once consumed their days, such as hospital visits, inhalation therapy, and emergency care, began to fade into the background. Emotional and physical exhaustion lifted gradually, replaced by moments of joy and reconnection. One participant recalled the striking change in her father’s health, reflecting:

*“Before transplantation, my dad was constantly inhaling, and I remember he was always coughing and always sick. Now he doesn’t inhale as often, and the coughing has disappeared. I have a different and healthier dad.”* (P4)

Another participant described how such changes in everyday activities symbolised much more:

*“He can now work, climb stairs without coughing or needing to rest after every step, and no longer relies on constant intravenous (IV) antibiotics, and we have a social life, the things that stopped when he was on the waiting list. This second chance brought our family renewed hope and joy.”* (P13)

Yet, alongside the gratitude, participants also expressed a range of complex emotions. Some spoke of persistent, quiet guilt tied to the knowledge that another person had to die for their loved one to live:

*“Pushing it away, I cannot handle it. Someone with healthy lungs needs to die.”* (P4)

*“I had a bad conscience and anxiety because I so desperately wanted someone to die so that my mother could live. However, at the same time, I did not want anyone else to die. So, processing that conflict was very difficult for me … I was 13 years old.”* (P17)

Despite the dramatic improvements in physical health and daily routines, these reflections revealed that the emotional impact of transplantation was layered and largely unresolved, especially among younger family members, who struggled to process the experience.

### Unmet support and informational needs before and after transplantation

Participants described a persistent lack of emotional and informational support across the transplantation process. Many felt that their caregiving roles and psychological strain were overlooked by healthcare professionals, leaving them unprepared for both crises and long-term demands. One participant emphasised this need for both clearer information and better support:

*“I wish the CF centre had given more information about what could actually happen, and that we as a family would need support even afterwards, because it isn’t easy afterwards.”* (P14)

The information provided was often outdated or insufficient, failing to reflect the complexities of recovery. In the face of these gaps, participants expressed a strong desire to connect with others in similar situations to share experiences and gain reassurance.

#### A critical need for information and comprehensive emotional support

Participants consistently described a lack of emotional and psychological support, not only during the transplantation procedure but across the entire trajectory of illness before, during, and after transplantation.

Many felt emotionally overwhelmed, unsupported, and left to manage distressing experiences alone. This absence of care left lasting emotional scars and contributed to long-term exhaustion and burnout. The need for clear, timely, and consistent information was also described as vital but often unmet. One participant shared how the absence of emotional support affected her long after the procedure, recalling:

*“I wasn’t offered any support during the process, and even several years after the transplantation, I still have nightmares of finding my partner drowned in blood. I suppressed my feelings for so long that I finally realised I needed to talk to someone, as the emotional weight left me feeling burnt out and collapsed.”* (P1)

Another participant expressed a deep longing for even minimal contact or acknowledgement from healthcare providers, saying:

*“I longed for a call from the CF clinic. It would have meant so much for me if they had checked if I needed help.”* (P18)

These unmet needs persisted even after the transplantation. For some, emotional exhaustion resurfaced years after the transplantation. One participant reflected:

*“A couple of years after the lung transplantation, I felt the need to create some distance from my [lung transplanted] father and the constant caregiving responsibilities.”* (P3)

Alongside these emotional gaps, participants also highlighted the inconsistency and inadequacy of information provided by both the CF teams and the transplantation centres. While some received written materials, they often found them outdated or insufficient to prepare families for the realities of recovery and long-term care.

*“I wish we had received more consistent information throughout the transplantation process and afterwards. New challenges arise after transplantation, but the healthcare system did not inform us about these. It was a complete shock.”* (P13)

#### A desire to connect with other families involved in the transplantation process

Beyond seeking professional support, participants expressed a strong desire to connect with others who had had similar experiences with transplantation. The emotional demands of the waiting period, combined with uncertainty and isolation, made the need for peer support particularly urgent.

Participants believed that sharing experiences with others who truly understood the challenges could offer both emotional relief and practical insight. These connections were described not just as helpful, but as something they deeply longed for. One participant reflected on the isolation of waiting, explaining that

*“Waiting was the toughest time. I really wish I had the opportunity to talk to someone who had already been through it.”* (P11)

Another participant highlighted the value of peer connections for emotional support and practical insights into the transplantation process:

*“I am willing to share my own experiences, as this extreme situation is truly understood only by those who have lived it. I think speaking with others would be very helpful, so families can know what to expect during the transplantation process.”* (P3)

## Discussion

This study explores the experiences of family members of individuals with CF who have undergone lung transplantation. The findings highlight a complex interplay of hope, fear, and emotional strain throughout the transplantation process. The inherent unpredictability of the transplantation process amplified this burden as families managed caregiving responsibilities, practical tasks, and ongoing uncertainty.

Many felt marginalised and insufficiently acknowledged by healthcare professionals. Addressing these challenges is essential for improving the well-being of both individuals with CF and their family members and should be a priority for healthcare providers, policymakers, and support systems.

A key finding was the lack of psychological and emotional support available for family members, particularly during the waiting period. Family members reported overwhelm, confusion, and isolation. These experiences echo the accounts of individuals with CF themselves, as described by Skogeland et al. (2025), and reflect broader systemic gaps in coordinated family support, as also noted by Brémault-Phillips et al. (2016) in families managing chronic illness. Such gaps can profoundly affect both patients’ and caregivers’ well-being.

The waiting period was characterised by fear, isolation, and moral conflict, consistent with earlier research by Glaze et al. (2021), Hansen et al. (2024), and Yagelniski et al. (2020). Family members described the emotional toll of hoping for a donor organ while being acutely aware that the fulfilment of this hope depended on someone else’s death. This duality of hope entwined with guilt has been previously described by Ivarsson et al. (2014) as both a gift and an existential burden.

Caregiving demands also affected family dynamics. Partners described emotional distance, while siblings and children often felt neglected due to the constant focus on the individual with CF. These experiences are consistent with research showing that caregivers frequently experience anxiety, helplessness, and burnout (Goldbeck et al., 2014; Yagelniski et al., 2020). This burden is intensified by the challenges of navigating healthcare systems, especially during lung transplantation (Daly et al., 2022).

The struggle to balance caregiving with everyday life underscores the urgent need for structured, tailored support for family caregivers, especially as healthcare systems increasingly rely on their involvement (Methi et al., 2024). Jesse et al. (2021) emphasise the crucial role of family caregivers in transplantation and advocate for the routine assessment of caregiver well-being, along with the provision of appropriate psychosocial resources to alleviate emotional strain.

Considering the emotional toll and psychosocial strain reported by family members during the transplantation process, these findings also support the European Cystic Fibrosis Society (ECFS) Standards of Care, which recommend routine mental health screening throughout the CF care continuum (Burgel et al., 2024). Screening during high-stress periods, such as transplantation, can help identify families in need of support. Its absence may explain why many felt overlooked, underscoring the importance of timely, coordinated interventions from CF nurses and psychosocial teams.

Furthermore, the burden of caregiver responsibilities can negatively affect patient outcomes, as noted by Myaskovsky et al. (2012), underscoring the need for targeted support. This includes assistance in navigating difficult topics such as end-of-life conversations. A notable finding from this study was the varied ways in which families approached discussions about death. While some found relief in confronting uncertainty, others avoided these sensitive conversations to preserve hope and emotional stability. These patterns are consistent with findings by Xu et al. (2024) in families of patients with advanced cancer, where responses ranged from open dialogue to protective silence.

This diversity in communication approaches illustrates that no single strategy is universally effective. Instead, such conversations are shaped by each family’s emotional capacity, values, and coping strategies. Healthcare professionals, particularly CF nurses, can play a pivotal role by facilitating structured conversations, as recommended by Glajchen et al. (2022), to create safe spaces for emotionally sensitive discussions and shared decision-making. However, stigma surrounding palliative care often complicates these conversations because such care is frequently misinterpreted as a sign of giving up (Bandieri et al., 2023).

Integrating palliative care into the transplantation process may address these challenges. Contrary to common misconceptions, palliative care is not limited to end-of-life scenarios but is beneficial throughout the course of serious illness. As emphasised by the World Health Organisation (WHO, 2024) and Sawatzky et al. (2016), palliative care aims to enhance quality of life by addressing the physical, emotional, and spiritual needs of both patients and their families. The Transplant Palliative Care Clinic model described by Wentlandt et al. (2016) demonstrates how such care can be effectively integrated into transplantation programmes, offering continuous support to patients and caregivers. Early integration of palliative care can enhance caregivers’ psychological resilience and better address their emotional, psychological, and practical needs, contributing to more person- and family-centred care (Gustafson & Song, 2020; Nolley & Morrell, 2021).

### Clinical implications

Given their close involvement in patient care, nurses and other healthcare professionals are uniquely positioned to identify family needs early and provide timely, person- and family-centred interventions. Supporting families through open communication, including discussions about palliative care, is essential to address the complex emotional and psychological burden surrounding the transplantation process.

Developing structured support programmes informed by clinical expertise and caregiver experiences can significantly enhance outcomes for both individuals with CF and their families. This implication aligns with the ECFS Standards of Care, which advocate for integrated psychosocial services and routine mental health screening, especially during high-stress periods such as transplantation (Burgel et al., 2024).

To address these needs, it is essential to develop evidence-based interventions explicitly tailored to transplantation caregivers. Such a programme should be carefully designed with attention to timing, content, and delivery format to ensure they are both accessible and impactful (Jesse et al., 2021). Nurses and healthcare professionals play a central role in implementing these interventions, fostering person- and family-centred care throughout the transplantation process.

Future research should investigate organisational and system patterns that influence the delivery of person and family- centred care, including communication practices within CF and transplantation care. Such insights can inform policy development and strengthen alignment with the ECFS Standards of Care.

### Strengths and limitations

A notable strength of this study is the high participation rate; nearly all invited consented, with only one declining to take part. Furthermore, family members reported consistent experiences and needs, regardless of the time elapsed since the lung transplantation. This includes family members of individuals who received transplants recently as well as those interviewed more than fifteen years after transplantation, suggesting that certain psychosocial challenges have persisted across time and disease stages.

However, several limitations should be considered. First, our study did not include bereaved family members of persons who died while on the waiting list or after lung transplantation, and their information and support needs may differ in important ways. Thus, the findings may not fully reflect the experiences of this group. Second, the retrospective nature of the study may have introduced recall bias as family members might not accurately remember past experiences, especially given the emotional intensity associated with lung transplantation (Althubaiti, 2016).

Third, the dual role of the first author (US) as both interviewer and CF nurse may have introduced social desirability bias. In response, several strategies aligned with qualitative research standards set out by Polit and Beck (2021) were employed to ensure rigour and trustworthiness. Reflexivity was maintained throughout the research process, with the author actively reflecting on her positionality and potential influence. Confidentiality was emphasised to foster a safe environment for open and honest disclosure. Finally, data interpretation was conducted collaboratively within the research team, strengthening the confirmability and dependability of the findings.

## Conclusion

This study highlights the complex psychological and emotional challenges faced by families of individuals with CF undergoing lung transplantation. The family members’ experiences revealed feelings of fear, isolation, and uncertainty, as well as a lack of sufficient support from healthcare systems. These insights underscore the importance of recognising and addressing family caregivers’ needs throughout the transplantation process. Future research should further explore how tailored interventions can best support families during these critical periods.

## Supplementary Material

Supplementary material COREQ_checklist

## Data Availability

The data underlying this study’s findings can be obtained from the corresponding author upon a reasonable request.
