# Supplementary material for: Balancing hope and uncertainty: family perspectives on lung transplantation in cystic fibrosis – a qualitative study
Source: Int J Qual Stud Health Well-being. 2026 Jan 24;21(1):2620417. doi: 10.1080/17482631.2026.2620417 (PMC12833888; doi:10.1080/17482631.2026.2620417)
Supplement: Supplementary material — COREQ_checklist [file ZQHW_A_2620417_SM6492.docx]

Consolidated criteria for reporting qualitative studies (COREQ): 32-item checklist

Please indicate in which section each item has been reported in your manuscript. If you do not feel an item applies to your manuscript, please enter N/A.

For further information about the COREQ guidelines, please see Tong *et al.*, 2017: <https://doi.org/10.1093/intqhc/mzm042>

| **No.** | **Item** | **Description** | **Section #** |
| --- | --- | --- | --- |
| **Domain 1: Research team and reflexivity** | | | |
| Personal characteristics | | | |
| *1.* | Interviewer/facilitator | Which author/s conducted the interview or  focus group? | US |
| *2.* | Credentials | What were the researcher's credentials? *E.g.*  *PhD, MD* | US: RN, MsC  IdM: MD, PhD  TG: RN, PhD |
| *3.* | Occupation | What was their occupation at the time of the  study? | US: RN, PhD student  IdM: MD, Researcher  TG: Professor |
| *4.* | Gender | Was the researcher male or female? | Female |
| *5.* | Experience and  training | What experience or training did the researcher  have? | US has undergone systematic training in qualitative research methods.  TG has undergone systematic training in qualitative research methods and has substantial experience in conducting qualitative studies. She also serves as a senior lecturer in a PhD course on thematic analysis.  IdM: Considerable experience in quantitative research regarding cystic fibrosis. |
| Relationship with participants | | | |
| *6.* | Relationship  established | Was a relationship established prior to study  commencement? | The relationship was established with some participants prior to the commencement of the study. |
| *7.* | Participant knowledge of the interviewer | What did the participants know about the researcher? *E.g. Personal goals, reasons for*  *doing the research* | Some participants knew US as an RN at one of the CF centers. |
| *8.* | Interviewer characteristics | What characteristics were reported about the interviewer/facilitator? *E.g. Bias, assumptions,*  *reasons and interests in the research topic* | US is interested in family members of individuals with CF who have undergone lung transplantation as an RN. The entire research group shares this interest. The author US dual role as both interviewer and CF nurse for some families may have led to social desirability bias. To address these limitations, strategies were implemented to enhance trustworthiness, including maintaining reflexivity, emphasizing confidentiality, and involving a multidisciplinary team in data analysis |
| **Domain 2: Study design** | | | |
| Theoretical framework | | | |
| *9.* | Methodological orientation and theory | What methodological orientation was stated to underpin the study? *E.g. grounded theory, discourse analysis, ethnography,*  *phenomenology, content analysis* | Reflexive Thematic Analysis. |
| Participant selection | | | |
| *10.* | Sampling | How were participants selected? *E.g. purposive,*  *convenience, consecutive, snowball* | Purposive sampling. |
| *11.* | Method of approach | How were participants approached? *E.g. face-*  *to-face, telephone, mail, email* | All presumptive participants were contacted via telephone or mail, where they were informed about the study's aim and provided with written information. |
| *12.* | Sample size | How many participants were in the study? | 19 |
| *13.* | Non-participation | How many people refused to participate or  dropped out? What were the reasons for this? | One individual declined participation without giving any reason. There were no dropouts. |
| Setting | | | |
| *14.* | Setting of data  collection | Where was the data collected? *E.g. home, clinic,*  *workplace* | They were allowed to choose both the timing and location for the interviews. These interviews were conducted in a quiet room at the hospital (n=3), via phone calls (n=3) or through or the digital Teams platform (n=13). |
| *15.* | Presence of non-  participants | Was anyone else present besides the  participants and researchers? | To the interviewer’s knowledge, no one else was present besides the participants and researchers. |

| *16.* | Description of sample | What are the important characteristics of the  sample? *E.g. demographic data, date* | The participants (family members) are described in detail in the manuscript. |
| --- | --- | --- | --- |
| Data collection | | | |
| *17.* | Interview guide | Were questions, prompts, guides provided by  the authors? Was it pilot tested? | Yes, a semi-structured interview guide was developed specifically for this study by US and TG. It was reviewed by healthcare professionals with experience in CF care (a nurse, a social worker, and a psychologist), as well as individuals with CF who had undergone lung transplantation and a participating family member. Based on their feedback, the guide was revised before use. The guide was also pilot-tested to ensure clarity and relevance, but pilot interviews were not included in the final analysis. |
| *18.* | Repeat interviews | Were repeat interviews carried out? If yes, how  many? | No |
| *19.* | Audio/visual recording | Did the research use audio or visual recording  to collect the data? | Yes, we used an audio recorder. |
| *20.* | Field notes | Were field notes made during and/or after the  interview or focus group? | No additional field notes were taken. |
| *21.* | Duration | What was the duration of the interviews or  focus group? | The interviews lasted between 26 to 87 minutes |
| *22.* | Data saturation | Was data saturation discussed? | Data saturation was not discussed, as it is not applicable within reflexive thematic analysis. |
| *23.* | Transcripts returned | Were transcripts returned to participants for  comment and/or correction? | No |
| **Domain 3: analysis and findings** | | | |
| Data analysis | | | |
| *24.* | Number of data  coders | How many data coders coded the data? | Two researchers (US and TG) coded the data. During familiarization, US read each interview multiple times, noting impressions and highlighting relevant text. US and TG collaboratively generated codes by identifying patterns aligned with the study’s objectives. Similar codes were grouped into themes and subthemes, emphasizing shared family experiences. Excel was used to organize codes by facilitating pattern detection. US and TG refined the themes by reassessing their consistency with the data. All themes were reviewed for coherence and distinctiveness. Finally, all authors discussed the themes to reach consensus. |
| *25.* | Description of the  coding tree | Did authors provide a description of the coding  tree? | A coding tree was described through the analytical process, showing how codes were grouped into themes and subthemes; however, no visual representation was provided. |
| *26.* | Derivation of themes | Were themes identified in advance or derived  from the data? | The themes were not identified in advance but derived from the data. |
| *27.* | Software | What software, if applicable, was used to  manage the data? | Excel and Microsoft Word. |
| *28.* | Participant checking | Did participants provide feedback on the  findings? | No |
| Reporting | | | |
| *29.* | Quotations presented | Were participant quotations presented to illustrate the themes / findings? Was each  quotation identified? *E.g. Participant number* | Yes. All identifiers were removed from the transcribed interviews and pseudonymized with a code to ensure that interviewes could not be identified. |
| *30.* | Data and findings  consistent | Was there consistency between the data  presented and the findings? | The authors attempted to keep the findings close to the original data, and the interviewer recognized the discussions in the results presentation. |
| *31.* | Clarity of major  themes | Were major themes clearly presented in the  findings? | The major themes are described in the results. |
| *32.* | Clarity of minor  themes | Is there a description of diverse cases or  discussion of minor themes? | The subthemes are described in the results. |

When submitting your manuscript via the online submission form, please upload the completed checklist as a Figure/supplementary file.

If you would like this checklist to be included alongside your article, we ask that you upload the completed checklist to an online repository and include the guideline type, name of the repository, DOI and license in the *Data availability* section of your manuscript.

Developed from: Allison Tong, Peter Sainsbury, Jonathan Craig, Consolidated criteria for reporting qualitative research (COREQ): a 32-item checklist for interviews and focus groups, International Journal for Quality in Health Care, Volume 19, Issue 6, December 2007, Pages 349–357, <https://doi.org/10.1093/intqhc/mzm042>
